# Analysis and verification of the circRNA regulatory network RNO_CIRCpedia_ 4214/RNO-miR-667-5p/Msr1 axis as a potential ceRNA promoting macrophage M2-like polarization in spinal cord injury

**DOI:** 10.1186/s12864-023-09273-w

**Published:** 2023-04-05

**Authors:** Jian Cao, Chongzhi Pan, Jian Zhang, Qi Chen, Tao Li, Dingwen He, Xigao Cheng

**Affiliations:** 1 Department of Orthopedics, The Second Affiliated Hospital of Nanchang University, Nanchang, Jiangxi 330006 China; 2 Institute of Orthopedics of Jiangxi Province, Nanchang, Jiangxi 330006 China; 3 Institute of Minimally Invasive Orthopedics, Nanchang University, Jiangxi, 330006 China; 4 Jiangxi Key Laboratory of Intervertebral Disc Disease, Nanchang University, Jiangxi, 330006 China; 5 Department of Orthopedics, The Second Affiliated Hospital of Nanchang University, 1 Minde Road, East Laker District, Nanchang, Jiangxi China

**Keywords:** Spinal cord injury, Circular RNAs, Immune inflammation, Macrophages, Polarization

## Abstract

**Background:**

CircRNAs are involved in the pathogenesis of several central nervous system diseases. However, their functions and mechanisms in spinal cord injury (SCI) are still unclear. Therefore, the purpose of this study was to evaluate circRNA and mRNA expression profiles in the pathological setting of SCI and to predict the potential function of circRNA through bioinformatics.

**Methods:**

A microarray-based approach was used for the simultaneous measurement of circRNAs and mRNAs, together with qPCR, fluorescence in situ hybridization, western immunoblotting, and dual-luciferase reporter assays to investigate the associated regulatory mechanisms in a rat SCI model.

**Results:**

SCI was found to be associated with the differential expression of 414 and 5337 circRNAs and mRNAs, respectively. Pathway enrichment analyses were used to predict the primary function of these circRNAs and mRNAs. GSEA analysis showed that differentially expressed mRNAs were primarily associated with inflammatory immune response activity. Further screening of these inflammation-associated genes was used to construct and analyze a competing endogenous RNA network. RNO_CIRCpedia_4214 was knocked down in vitro, resulting in reduced expression of Msr1, while the expression of RNO-miR-667-5p and Arg1 was increased. Dual-luciferase assays demonstrated that RNO_CIRCpedia_4214 bound to RNO-miR-667-5p. The RNO_CIRCpedia_4214/RNO-miR-667-5p/Msr1 axis may be a potential ceRNA that promotes macrophage M2-like polarization in SCI.

**Conclusion:**

Overall, these results highlighted the critical role that circRNAs may play in the pathophysiology of SCI and the discovery of a potential ceRNA mechanism based on novel circRNAs that regulates macrophage polarization, providing new targets for the treatment of SCI.

**Supplementary Information:**

The online version contains supplementary material available at 10.1186/s12864-023-09273-w.

## Introduction

Spinal cord injury (SCI) causes severe damage to parts of the central nervous system that incurs significant psychological and physical burdens for affected individuals and their families, with additional economic costs that impact society as a whole. An estimated 250,000–500,000 people throughout the world are estimated to suffer SCIs each year [1]. The SCI pathogenesis consists of two complex stages of primary and secondary damage. During the primary phase, the mechanical injury irreversibly damages the nerve tissues, whereafter secondary damage further exacerbates this injury through processes including apoptotic cell death, inflammatory activity, oxidative stress, and local microenvironmental inhibition, ultimately contributing to sustained losses in motor and sensory functionality [2]. A range of therapies have been employed for SCI patients, including stem cell transplantation, high doses of methylprednisolone, and surgical decompression, but outcomes in treated patients remain unsatisfactory [3, 4]. As the primary injury cannot be controlled or reversed in SCI patients, therapeutic efforts primarily center around counteracting the molecular mechanisms governing secondary injury through the identification of new targets associated with this deleterious process.

Over 98% of the human genomic sequences are non-coding RNAs (ncRNAs) [5]. While they do not produce proteins, these RNAs, which include species such as circular RNAs (circRNAs) and microRNAs (miRNAs), can control diverse biological processes under healthy and pathological conditions through transcriptional, translational, and epigenetic regulatory mechanisms, highlighting their potential relevance in the treatment of various diseases [6]. As covalently-linked closed-loop RNA structures, circRNAs lack traditional 3’ or 5’ ends and associated structural motifs, rendering them resistant to exonucleases such as RNaseR [6]. Many circRNAs are produced by eukaryotic genomes, and their expression is both time- and tissue-specific [5]. Mechanistically, these circRNAs can competitively bind certain miRNAs as competing endogenous RNAs (ceRNAs), resulting in indirect regulation of RNA expression [5]. Targeting circRNAs may thus represent an effective means of treating a range of diseases. Many reports have shown that circRNAs play important roles in various neurological conditions such as tumors of the CNS [7], cerebral ischemia [8], and neurodegeneration [9, 10]. For instance, Chen et al. reported that circPTN sponges miR-145-5p/miR-330-5p to promote proliferation and stemness in glioma [7]. Wang et al. reported that overexpression of circUCK2 attenuates neuronal apoptosis in cerebral ischemia–reperfusion injury via miR-125b-5p/GDF11 signaling [8]. Liu et al. reported that circ-Pank1 promotes dopaminergic neuron neurodegeneration through modulation of the miR-7a-5p/α-syn pathway in Parkinson’s disease [10].

Recently, several studies have analyzed the possible functions of circRNAs following SCI in rodent models using bioinformatics. For example, Ye et al. discovered that circRNA-7079, circRNA-7078, and circRNA-6777 have important roles in vascular endothelial proliferation, migration, and angiogenesis, suggesting that they may be potential therapeutic targets for SCI [11]. Tong et al. used bioinformatics prediction and preliminary in vitro verification to show that circ-Usp10 may target miR-152-5p to regulate CD84, resulting in microglial activation and neuronal death [12]. Thus, circ-Usp10 may be a potential therapeutic target for SCI. Wang et al. used a bioinformatic approach to predict the CircPlek/miR-135b-5p/TGF-βR1 axis, and further in vitro experiments confirmed its influence on SCI-associated fibrosis [13]. Nevertheless, the functions and regulatory mechanisms of circRNAs in SCI are poorly understood.

Here, a microarray approach was used to profile the expression of mRNAs and circRNAs in spinal cord tissue samples collected from a rat model 3 days after SCI. The functional roles of differentially expressed (DE) genes and associated pathways were then explored. In comparison with similar studies, the novelty of the present investigation is the construction of a ceRNA network that is closely related to immune inflammation in SCI. Together, these analyses offer new information regarding the molecular basis for SCI. Specifically, cell-based experiments confirmed that knockdown of RNO_CIRCpedia_4214 can promote M2 macrophage polarization and participate in the immune inflammatory reaction.

## Materials and methods

### Animals

The study used adult male Sprague–Dawley rats (200-240 g) that were fed under standard conditions (23–25 °C, 12 h light/dark cycle) with free access to food and water for at least one week before the experiment. The Animal Ethics Committee of the Second Affiliated Hospital of Nanchang University approved all animal studies, and the NIH guidelines for the care and use of laboratory animals were used to ensure that these rats received appropriate care. Additionally, the study was conducted in accordance with ARRIVE guidelines (https://arriveguidelines.org).

### SCI model establishment

An SCI model was established using the Allen strike technique [14]. Rats were anesthetized by isoflurane inhalation and randomly assigned to the sham and SCI groups (*n* = 3/group). A T10-level laminectomy was conducted to facilitate spinal cord exposure without disruption of the dura mater. SCI modeling at the T10 level was then performed using a modified Allen’s weight-drop apparatus (10 g × 80 mm; MEYUE, Changsha, China). Success was determined based on visual evidence of local spinal cord edema, lower limb twitching, and spastic wagging of the tail. Sham rats underwent the same laminectomy procedure without damage to the spinal cord. After surgery, surgical sutures were used to close the overlying muscle and cortical layers. Manual voiding of the bladder was conducted three times a day for all rats until spontaneous voiding activity had recovered.

### Microarray analyses

The expression patterns of mRNAs and circRNAs in the spinal cord in the 1.5-cm region surrounding the T10 level were investigated 3 days after SCI using an Agilent ceRNA microarray 2019 (8 * 60 K, design ID: 086243). A mirVana™ RNA Isolation Kit (AM1561) was used, according to the provided directions, to extract RNA from target samples, followed by quantification using a NanoDrop® 2000 (Thermo Scientific, Inc.) instrument. An Agilent Bioanalyzer 2100 (Agilent Technologies, Inc.) was used to assess RNA integrity. OEbiotech (Shanghai, China) performed all microarray hybridization analyses. Initially, the total RNA was reverse-transcribed to double-stranded cDNA, which then served as a template for cRNA synthesis with Cyanine 3 cytidine triphosphate (CTP) serving as a label. After labeling, the cRNA was hybridized to appropriate microarrays, which were then washed and scanned using an Agilent Scanner G2505C. The feature extraction software (v 10.7.1.1, Agilent Technologies) was used to extract the original data from the microarray image, and Genespring (v 14.8, Agilent Technologies) was then used for further analysis. The raw data were normalized with a quantile algorithm. Probes with over 75% flagged “Detected” samples in one group were selected for further investigation. Fold-change (FC) values and *P*-values (Student’s t-test) were used to identify DE genes (FC > 2.0, *P* < 0.05). Hierarchical cluster analysis of DEGs was performed using R (v 3.2.0) to evaluate gene expression in different groups and samples. All original data were uploaded to the GEO database (GSE210609).

### Functional enrichment analyses

Based on the hypergeometric distribution, the functional roles of both DE mRNAs and mRNAs that were identified as targets of circRNA-miRNA regulatory relationships were subjected to GO (Gene Ontology) and KEGG (Kyoto Encyclopedia of Genes and Genomes) pathway enrichment analyses. R (v 3.2.0) was used to draw the column diagram and bubble diagram of significantly enriched terms. The GO analysis classified mRNA enrichment into the cellular component (CC), biological process (BP), and molecular function (MF) categories (http://www.geneontology.org). KEGG analysis showed enrichment of the mRNAs in particular pathways (https://www.kegg.jp/kegg/kegg1.html). In addition, a gene set enrichment analysis (GSEA) was used to determine whether there were significant differences between pre-established gene sets in the SCI and sham groups. Briefly, the analysis used a predefined gene set, and the genes were ranked according to the degree of differential expression between the two sample types. The genes in the predefined set were then ranked according to their degree of enrichment (http://www.gsea-msigdb.org).

### CircRNA-miRNA-mRNA network construction

Functionally, circRNAs can act as ceRNAs to sequester specific miRNAs, thus altering downstream gene expression. To explore the potential interplay between DE circRNAs and DE mRNAs, the top 200 DE circRNAs and DE mRNAs were selected for co-expression analyses, with those pairs that were positively correlated with one another (Pearson’s *r* > 0.9, *P* < 0.05) being retained for additional analyses. miRanda was used for the identification of miRNA targets using default parameters (http://www.microrna.org/microrna/home.do). The ceRNA network was constructed by merging the common targeted miRNA and ceRNA scores based on the MuTaME method [15]. Target prediction of miRNAs to the aforementioned DE circRNAs and mRNAs was performed by using all known rat miRNAs in the database Mibase22. Cytoscape (v 3.7.2) was used to build a circRNA-micro (mi)RNA-mRNA interaction network.

### qPCR

qPCR was used to analyze the expression levels of the genes in the two groups. Total RNA was extracted from spinal cord tissue using TRIzol (Takara, Japan). The purity and quantity of the RNA were assessed on an ultraviolet spectrophotometer (SYNERGY H1, BioTek, USA) and the expression of circRNAs, mRNAs, and GAPDH were evaluated with PrimeScript RT Master Mix (Takara, Japan), SYBR Premix Ex Taq II kits (Takara, Japan), and a CFX96 qRT-PCR Detection System (Bio-Rad, USA). Levels of miRNAs and U6 expressed were measured with a bulge-Loop miRNA qRT-PCR Starter Kit (RiboBio, Guangzhou, China). The qPCR parameters were as follows: 95 °C for 30 s(pre-denaturation), followed by 40 cycles (amplification) of 95 °C for 15 s (denaturation), 60 °C for 30 s (annealing), and 60 °C for 30 s (extension). Experiments were performed in triplicate, with GAPDH as a control for mRNAs and circRNAs, while U6 served as a control for miRNAs. The 2^–ΔΔCT^ method was used for comparative quantitation. CircRNA covalent loop structures were confirmed through Sanger sequencing. The primers used are listed in Supplementary Table 1.

### Cell culture and transfection

RMa-bm macrophages were purchased from the Shanghai Chunmai Biotechnology Co., Ltd (Shanghai, China), and were cultured in RPMI-1640 (11875, Solarbio) containing 0.1% antibiotics (15070063, Gibco) and 10% FBS (10099141, Gibco) at 37 °C in a humidified atmosphere of 5% CO_2_. Macrophages were seeded in 6-well plates and grown to 30–50% confluence, at which time media were exchanged for antibiotic-free media. Cells were then transfected with siRNAs (RiboBio, Guangzhou, China) (70 nM) using the riboFECTCP transfection kit (C10511-05, RiboBio, Guangzhou, China). At 48 h post-transfection, macrophages were then treated for 18 h with LPS (1 µg/ml; Sigma-Aldrich, USA) [16].

### Fluorescence in situ hybridization (FISH)

The FISH kit (RiboBio Co.) was used for the analysis of RMa-bm macrophages using RNO_CIRCpedia_4214 probes (Supplementary Table 2) purchased from Servicebio (Wuhan, China). Cells were fixed with 4% paraformaldehyde for 20 min and permeabilized using 0.5% Triton X-100 for 10 min. The cells were washed three times in PBS, with 15 min per wash. The pre-hybridization solution was added to the cells and incubated at 37 °C for 1 h, followed by the hybridization solution with RNO_CIRCpedia_4214 probes labeled using Cy-3 (red) overnight at 37 °C. Nuclei were counterstained with 4′,6-Diamidino-2-phenylindole (blue). Fluorescence was examined using a Nikon Laser Scanning Confocal Microscope (Nikon Instruments, Japan).

### Dual-luciferase reporter assay

Dual-luciferase reporter gene assays were conducted using a kit (Promega, Wisconsin, USA) according to the provided instructions. Both wild-type (WT) and mutated (MUT) forms of RNO_CIRCpedia_4214 3′-UTR were inserted into dual-luciferase reporter plasmids by RiboBio (Guangzhou, China). The luciferase vectors were then transfected into cells along with miR-667-5p mimics or miR-NC. 100 nmol reporter plasmids (RNO_CIRCpedia_4214, RNO_CIRCpedia_4214 mut) together with miR-667-5p or negative control were transfected into 293-T cells. After 48 h of transfection, the dual-luciferase reporter system kit was used to detect the luciferase activity.

### Western immunoblotting

Total protein was isolated from macrophages and quantified by BCA assays, followed by separation on 10% SDS-PAGE (5% stacking gel) gels and transfer to PVDF membranes (0.45 μm micropore size). The blots were blocked with non-fat milk, followed by incubation overnight with anti-iNOS (Proteintech, 18985-1-AP, 1:1000), anti-Arg1 (Proteintech, 16001-1-AP, 1:40000), or anti-Alpha Tubulin (Proteintech, 11224-1-AP, 1:5000) at 4 °C. After washing, the membranes were probed at room temperature for 1.5 h with HRP-conjugated goat anti-rabbit IgG, washed using TBST, and proteins were detected with an Enhanced Chemiluminescent Kit (UE, Suzhou, China) and a TECAN luminescent imaging system.

### Statistical analysis

Data are presented as means ± standard deviation (mean ± SD) and were compared via Student’s t-tests. SPSS 20.0 (IBM Corp., Armonk, NY, USA) was used for all statistical analyses, with *P* < 0.05 as the significance threshold.

## Results

### Microarray-based analysis of SCI-related circRNA and mRNA expression

**Fig. 1:**
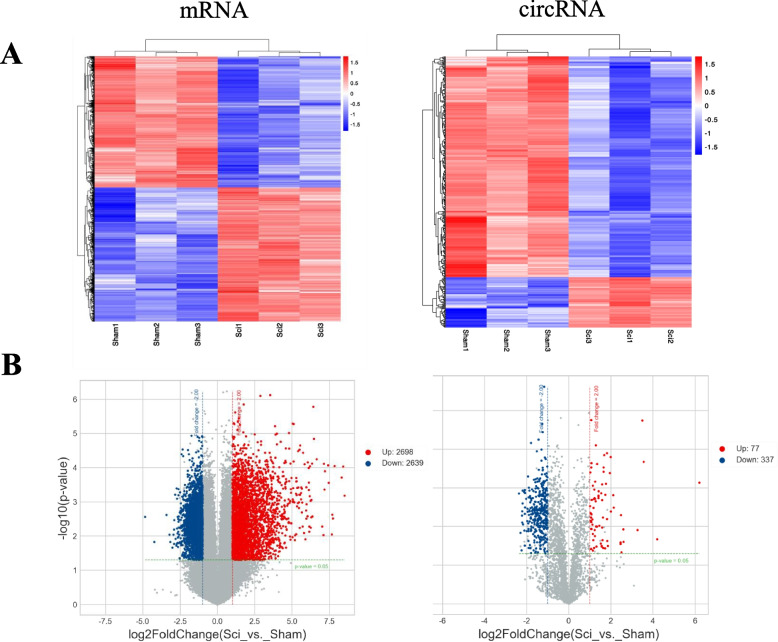
Expression profiles of circRNAs and mRNAs in the SCI and control groups. **A** Heatmaps showing the hierarchical clustering of differentially expressed circRNAs and mRNAs between the SCI and control groups. **B** Volcano plots showing the up-regulated and down-regulated circRNAs and mRNAs between the two groups. Up-regulated expression is indicated in red, and down-regulated expression is indicated in blue

Initial microarray analysis showed that 77 circRNAs and 2,698 mRNAs were upregulated in SCI model rats relative to the sham controls, while 337 circRNAs and 2,639 mRNAs were significantly downregulated (FC > 2.0, *P* < 0.05). These results were further represented with a two-dimensional hierarchical clustering heatmap (Fig. 1A), and volcano plots (Fig. 1B). The 10 most significantly up- and downregulated circRNAs and mRNAs in SCI are listed in Tables 1 and 2. The most upregulated mRNAs and circRNAs in SCI were Cd8a (log_2_FC = 8.5) and RNO_CIRCpedia_5625 (log_2_FC = 6.2), respectively, while the most downregulated were Serhl2 (log2FoldChange = −4.8) and RNO_CIRCpedia_5663 (log_2_FC = −2.3).

**Table 1:** The ten most significantly up- and down-regulated circRNAs

**ID**	**Gene**	**Regulation**	**log2(fold-change)**	***P*-value**
RNO_CIRCpedia_5625	Gpnmb	Up	6.201847527	0.000739415
RNO_CIRCpedia_9210	Diaph3	Up	4.201207596	0.021539086
RNO_CIRCpedia_2816	Abca1	Up	3.557176697	0.000212097
RNO_CIRCpedia_2817	Abca1	Up	3.496915685	0.000018100
RNO_CIRCpedia_9131	Mboat1	Up	3.267827551	0.012431732
RNO_CIRCpedia_9819	Mad2l1	Up	2.603245315	0.025138884
RNO_CIRCpedia_6416	Arhgap10	Up	2.594929004	0.011900882
RNO_CIRCpedia_2639	Mis18bp1	Up	2.502351265	0.046708952
RNO_CIRCpedia_6360	Diaph3	Up	2.480652711	0.005127482
RNO_CIRCpedia_7090	Fnip2	Up	2.465080882	0.026194612
RNO_CIRCpedia_9791	Dync1i1	Down	−2.117374259	0.001286605
RNO_CIRCpedia_2607	Akap6	Down	−2.174283052	0.008870334
RNO_CIRCpedia_4333	Elmod1	Down	−2.194557941	0.002536671
RNO_CIRCpedia_3125	Dync1i1	Down	−2.195002954	0.001192992
RNO_CIRCpedia_8234	Hecw1	Down	−2.211353352	0.007908662
RNO_CIRCpedia_3957	Slc9b1	Down	−2.215322409	0.004055335
RNO_CIRCpedia_3320	Far2	Down	−2.224097049	0.005259303
RNO_CIRCpedia_5	Cntnap5b	Down	−2.246843231	0.041411020
RNO_CIRCpedia_8719	Scn1a	Down	−2.293958841	0.014596644
RNO_CIRCpedia_5663	Prmt8	Down	−2.364926330	0.001397406

**Table 2:** The ten most significantly up- and down-regulated mRNAs

**ID**	**Gene**	**Regulation**	**log2(fold-change)**	***P*-value**
XM_008762976.2	Cd8a	Up	8.526989698	0.000667062
ENSRNOT00000071033	LOC100910371	Up	8.426124928	0.000092400
XM_006236630.3	Cd8a	Up	8.328621002	0.000194529
ENSRNOT00000083886	LOC100910371	Up	7.855782413	0.000089800
ENSRNOT00000077186	Lilrb4	Up	7.742890474	0.003027550
ENSRNOT00000004315	Upk1b	Up	7.708363769	0.002377905
XM_017592483.1	Apobec1	Up	7.672429292	0.008973345
ENSRNOT00000073646	AABR07030793.1	Up	7.467178552	0.000278452
ENSRNOT00000077158	LOC100909671	Up	7.424956005	0.000085700
XM_008768424.2	Cd300ld	Up	7.203848507	0.000126514
XM_006248003.3	Chodl	Down	−2.892597836	0.003411325
ENSRNOT00000001853	Oas1k	Down	−2.906983511	0.017150164
ENSRNOT00000039696	Iqcf3	Down	−2.908392067	0.006489217
XM_006255051.3	Car7	Down	−2.959090551	0.009163195
XM_017599644.1	Zfp385d	Down	−3.026445232	0.021710918
ENSRNOT00000002148	Hoxd10	Down	−3.160293641	0.014992648
ENSRNOT00000079473	Ryr1	Down	−3.221028608	0.009133346
ENSRNOT00000018529	Defa24	Down	−3.419354784	0.002440430
ENSRNOT00000048782	Oas1k	Down	−4.233555972	0.014955077
XM_017595059.1	Serhl2	Down	−4.844542134	0.002740572

### Functional annotation of differentially expressed mRNAs

**Fig. 2:**
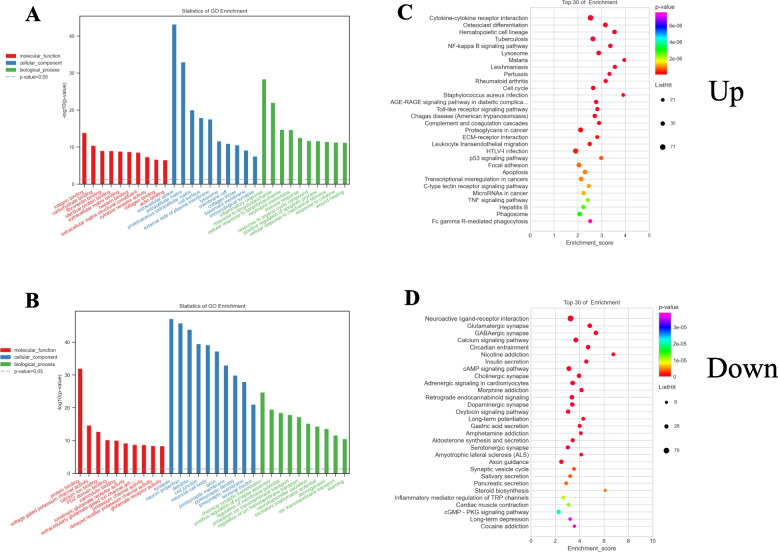
GO enrichment analysis (**A**, **B**) and KEGG pathway analysis (**C**, **D**) for the up- and downregulated differentially expressed mRNAs (**B**, **D**)

To gain insight into the functions played by differentially expressed mRNAs in SCI model rats, GO and KEGG enrichment analyses were performed. The most significantly upregulated DE mRNAs were associated with the GO BP, CC, and MF terms inflammatory response, extracellular space, and integrin binding, respectively (Fig. 2A), while the most significantly downregulated DE mRNAs were associated with the respective chemical synaptic transmission, synapse, and protein binding terms (Fig. 2B). Further KEGG analyses showed the most upregulated DE mRNAs to be enriched in the hematopoietic cell lineages, cytokine-cytokine receptor interactions, and osteoclast differentiation pathways, among others (Fig. 2C), while the most downregulated DE mRNAs were enriched in neuroactive ligand-receptor interactions, the glutamatergic synapse, and GABAergic synaptic pathways, among others (Fig. 2D).

**Fig. 3:**
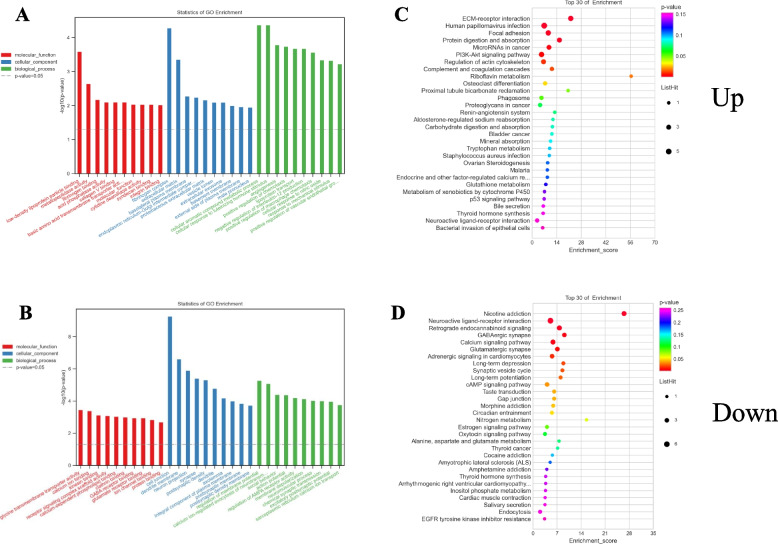
GO enrichment and pathway analysis for up- and downregulated mRNAs regulated by circRNA-miRNA. Top 10 enriched GO terms of up and down-regulated mRNAs regulated by circRNA-miRNA (**A**, **C**). KEGG pathway analysis of up- and down-regulated mRNAs regulated by circRNA-miRNA in SCI (**B**, **D**)

**Fig. 4:**
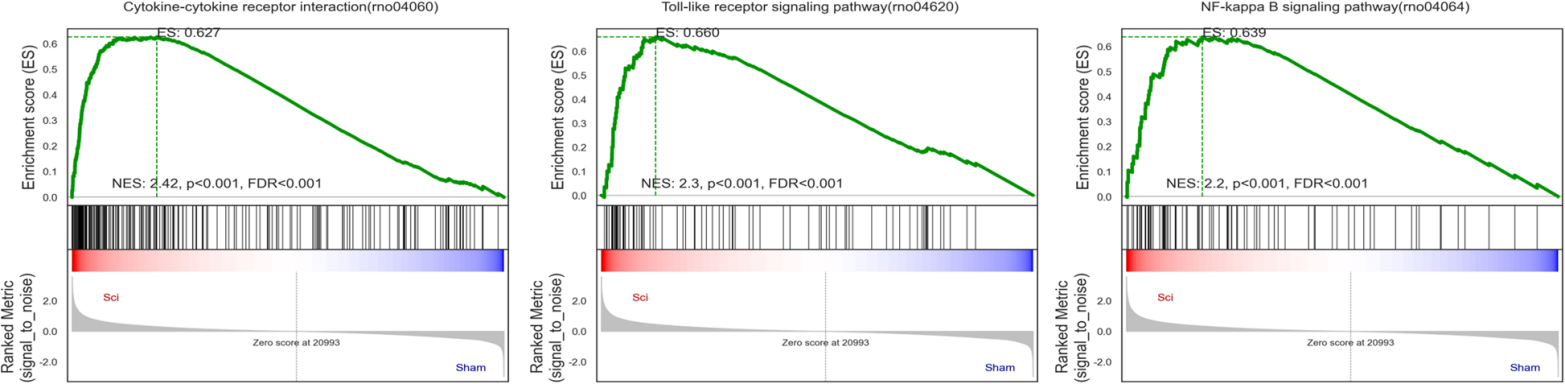
Gene Set Enrichment analysis (GSEA) for mRNAs

Mechanistically, circRNAs can function as ceRNAs by sequestering specific miRNAs and thereby indirectly controlling gene expression. The top 200 up- and downregulated circRNAs and mRNAs that were dysregulated following SCI modeling were identified, revealing 379 circRNA-miRNA-mRNA pairs. A complete ceRNA network incorporating 39 circRNAs, 37 miRNAs, and 122 mRNAs was constructed. The DE mRNAs in this network were then further analyzed, with GO analyses showing that upregulated DE mRNAs were enriched in the cellular aromatic compound metabolic process (BP), fibrinogen complex (CC), and low-density lipoprotein particle binding (MF) terms (Fig. 3A), while the most downregulated DE mRNAs were enriched in the regulation of membrane potential (BP), cell junction (CC), and glycine transmembrane transporter activity (MF) terms (Fig. 3B). KEGG pathway analyses further indicated that the upregulated DE mRNAs were enriched in 30 pathways including ECM-receptor interactions, focal adhesion, and the PI3K-Akt signaling pathway (Fig. 3C), while downregulated DE mRNAs were enriched in the nicotine addiction, neuroactive ligand-receptor interaction, and retrograde endocannabinoid signaling pathways, among others (Fig. 3D). GSEA was used to evaluate all mRNAs in the microarray to identify the top 15 most upregulated pathways in SCI model rats (Supplementary Table 3). This showed that these pathways were closely linked to the inflammatory immune response, with enrichment for the cytokine-cytokine receptor interaction (rno04060), Toll-like receptor signaling (rno04620), and NF-kappa B signaling (rno04064) pathways (Fig. 4).

### qPCR-based verification of microarray results

**Fig. 5:**
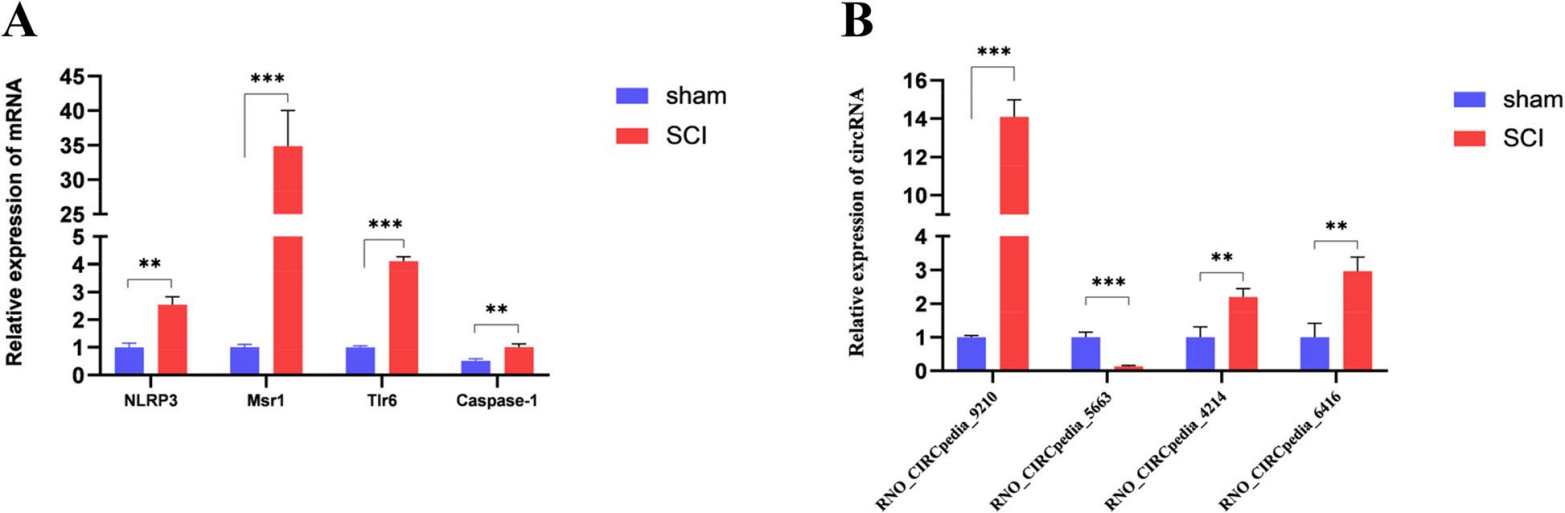
Verification of differentially expressed mRNAs (**A**) and circRNAs (**B**) by qPCR in the SCI group (*n* = 3). The data are presented as the mean ± SD (*n* = 3). *P < 0.05; **P < 0.01; ***P < 0.001, compared with the sham group (*n* = 3) (Student’s t-test)

To verify the microarray findings, four mRNAs (NLRP3, Msr1, Tlr6, Caspase-1) and four circRNAs (RNO_CIRCpedia_9210, RNO_CIRCpedia_6416, RNO_CIRCpedia_4214, RNO_CIRCpedia_5663) were selected at random for qPCR-based verification (Fig. 5A, B). These results confirmed the findings of the microarray analyses, thus reaffirming the reliability of these results.

### Immune inflammation-related ceRNA network establishment

**Fig. 6:**
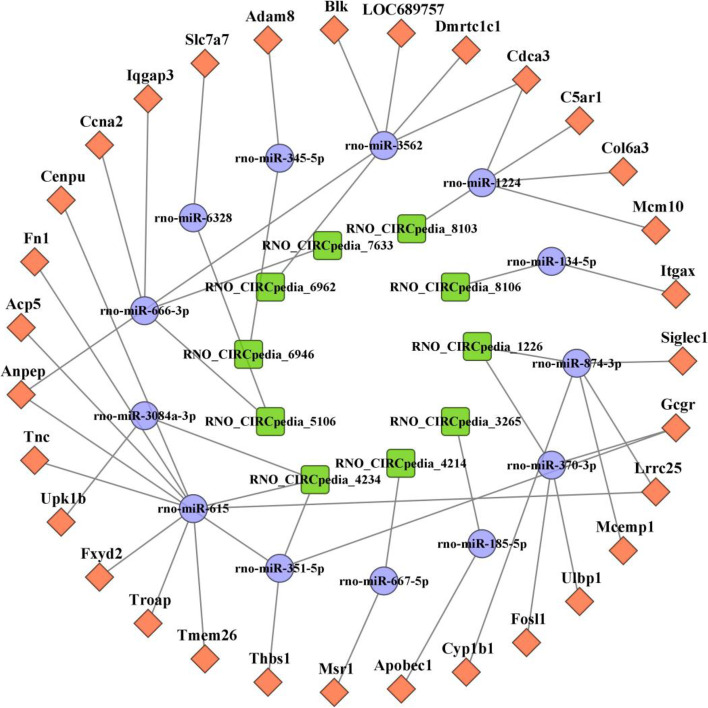
Construction of the circRNA-miRNA-mRNA ceRNA network based on selected genes related to immune and inflammation. The ceRNA regulatory network includes 31 mRNAs, 13 miRNAs and 10 circRNAs. The orange rectangular nodes represent mRNAs, the blue circular nodes indicate miRNAs, and green rectangular nodes denote circular RNAs

To further explore the role of immune-mediated inflammatory activity within the spinal cord following SCI, mRNAs identified as associated with inflammatory and immune GO and KEGG terms were selected, leading to the identification of 31 upregulated DE mRNAs (> 2 FC, *P* < 0.05) including C5ar1, Cdca3, Tnc, and Msr1. Then, circRNA-miRNA-mRNA networks were constructed for these mRNA targets, resulting in the establishment of a network composed of 31 mRNAs, 13 miRNAs, and 10 circRNAs (Fig. 6). This network suggested that RNO_CIRCpedia_8103, RNO_CIRCpedia_8106, RNO_CIRCpedia_1226, RNO_CIRCpedia_3265, RNO_CIRCpedia_4214, RNO_CIRCpedia_4234, RNO_CIRCpedia_5106, RNO_CIRCpedia_6946, RNO_CIRCpedia_6962, RNO_CIRCpedia_7633 may act as ceRNAs to control the expression of the identified immunity and inflammation-associated mRNAs, thereby shaping the pathogenesis of SCI.

### RNO_Circpedia_4214 controls the polarization of macrophages

**Fig. 7:**
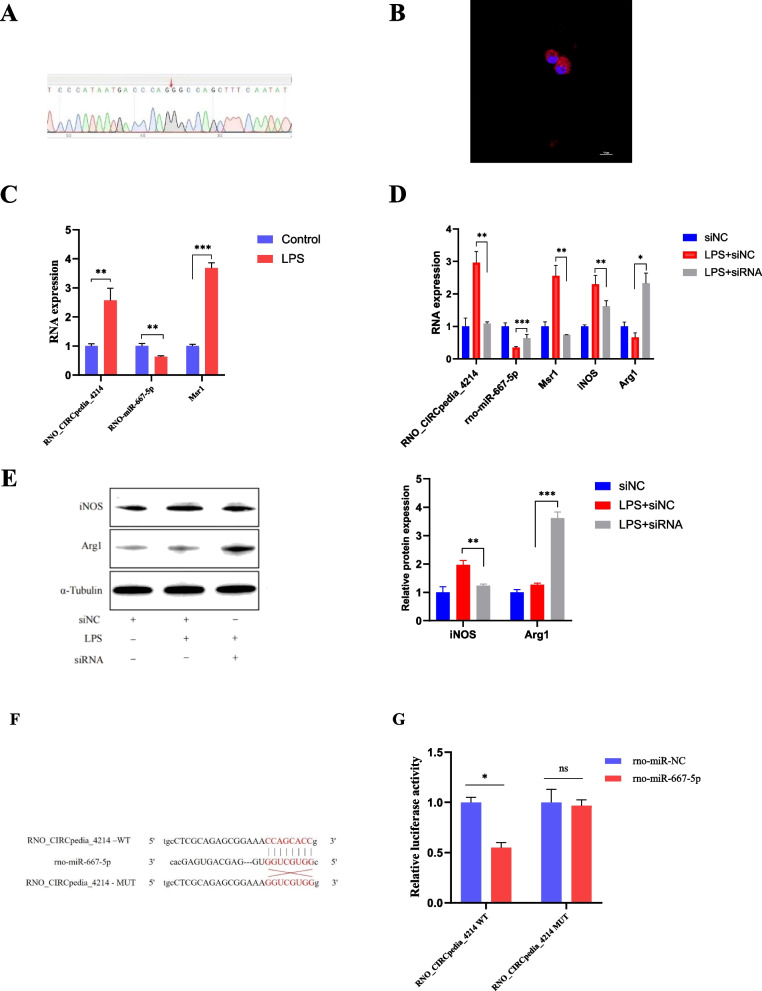
RNO_CIRCpedia_4214 facilitated macrophage polarization to the M2 type. **A** Sanger sequencing confirmed the back-splice junction sites of RNO_CIRCpedia_4214. **B** RNA FISH showed that RNO_CIRCpedia_4214 was predominantly localized in the cytoplasm. RNO_CIRCpedia_4214 probes were labeled with Cy-3. The nuclei were stained with DAPI. Scale bar, 10 µm. **C**–**E** The expression levels of RNO_CIRCpedia_4214, rno-miR-667-5p, Msr1, iNOS and Arg1 mRNA or protein were detected by RT-qPCR and western blotting, respectively, in macrophages transfected with siRNA, as well as controls. **F** The putative binding sequences between RNO_CIRCpedia_4214 and RNO-miR-667-5p. **G** Dual-luciferase reporter assays were performed to verify whether RNO-miR-667-5p was a target of RNO_CIRCpedia_4214. The data are presented as the mean ± SD (*n* = 3). *P < 0.05; **P < 0.01; ***P < 0.001

To verify the roles of the identified circRNAs as regulators of immune-mediated inflammatory activity, the RNO_CIRCpedia_4214/rno-miR-667-5p/Msr1 axis was selected for in vitro evaluation. Initial Sanger sequencing confirmed that RNO_CIRCpedia_4214 contained a back-spliced junction consistent with its circular nature (Fig. 7A), and RNA-FISH indicated that this circRNA primarily localized to the cytoplasm (Fig. 7B). qPCR confirmed that after LPS treatment, macrophages upregulated both RNO_CIRCpedia_4214 and Msr1 (Fig. 7C). Lower levels of RNO-miR-667-5p expression in macrophages were also seen after LPS treatment (Fig. 7C). After RNO_CIRCpedia_4214 knockdown, lower levels of the circRNA were seen in macrophages, confirming the efficiency of siRNA transfection, with a corresponding drop in Msr1 and iNOS expression, while RNO-miR-667-5p and Arg1 levels increased in the cells (Fig. 7D). Western immunoblotting also indicated that RNO_CIRCpedia_4214 silencing promoted macrophage polarization toward the M2 subtype in comparison with control macrophages (Fig. 7E). Figure 7F shows the putative binding sites between RNO_CIRCpedia_4214 and rno-miR-667-5p. In addition, dual-luciferase assays indicated that RNO_CIRCpedia_4214 could bind to rno-miR-667-5p (Fig. 7G).

## Discussion

**Fig. 8:**
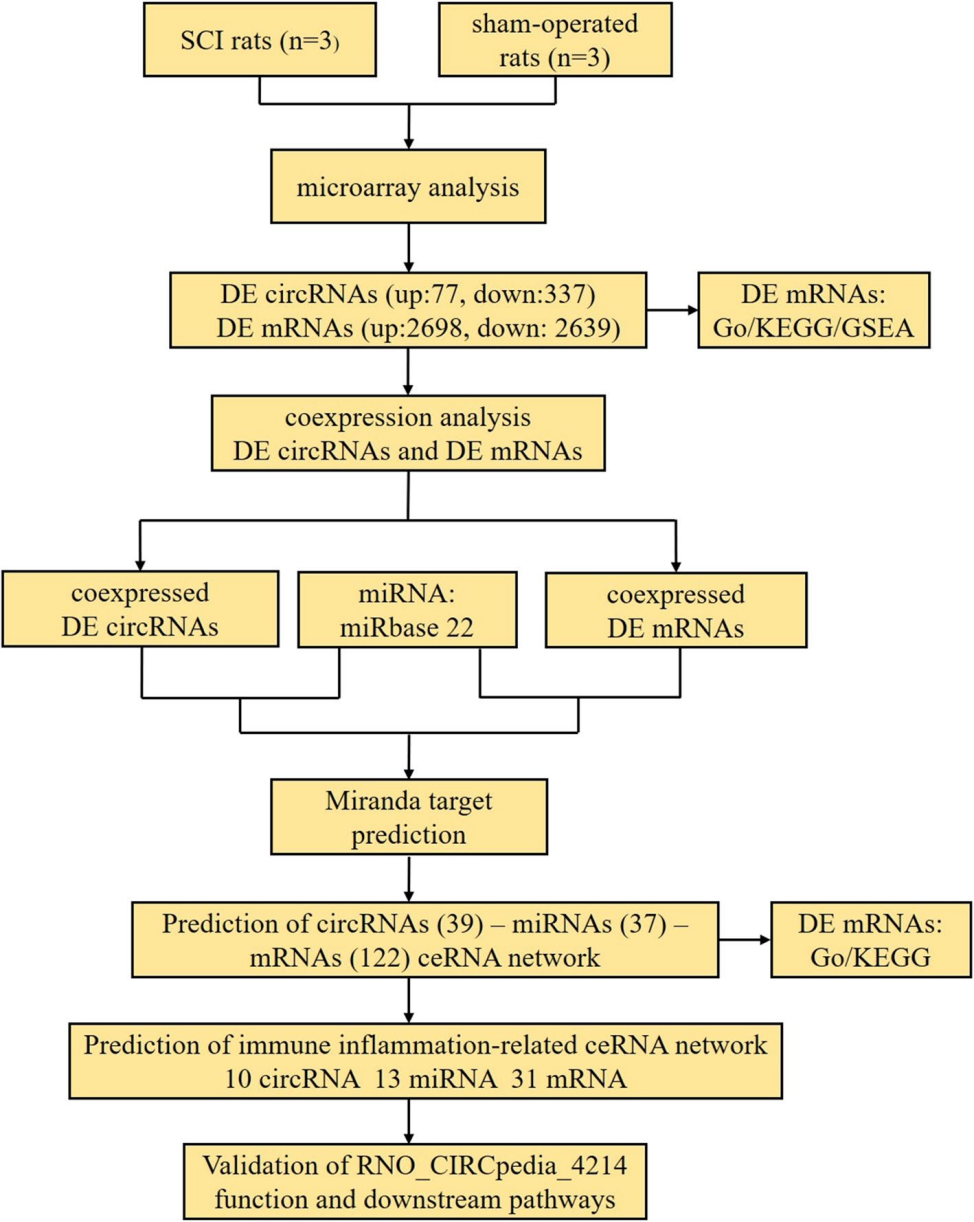
The flow chart of this study

There is substantial evidence supporting the ability of circRNAs to influence both normal growth and development as well as pathological processes through mechanisms linked to oxidative stress, proliferation, inflammation, autophagy, apoptosis, and differentiation, among others [17–19]. Efforts to clarify how circRNAs shape the pathogenesis of SCI have the potential to develop treatments for this debilitating condition; however, the information on this topic is relatively limited. Experimental methods for circRNA prediction are still expensive and time-consuming. Therefore, various bioinformatic computational models for predicting circRNA-disease associations have been developed [20–22], such as network algorithm-based and machine learning-based models and the LLCDC method, for the accurate and efficient screening of circRNAs associated with diseases. In this study, we explored circRNA closely related to SCI in rats by microarray and bioinformatics analyses. CircRNAs and mRNAs that were dysregulated in a rat model of SCI were investigated, leading to the identification of 5337 DE mRNAs and 414 DE circRNAs in SCI model rats relative to control animals. As the immune inflammatory response forms a major part of the secondary SCI injury, we analyzed the genes involved in these pathways in detail and constructed the ceRNA network, hoping to provide a new therapeutic direction and molecular basis for the treatment of SCI. Figure 8 shows the flow-chat of the present study.

The roles of the identified DE mRNA were investigated using GO and KEGG analyses. Upregulated DE mRNAs were associated with GO terms such as fibrin junctions, extracellular matrix composition, cytokine receptor activity, inflammatory immune response, and apoptosis, while downregulated DE mRNAs were associated with ion channel activity, neuronal projections, and positive regulation of synapse assembly. KEGG analyses also revealed these DE mRNAs to be enriched in the phospholipase D, retrograde endocannabinoid, and calcium signaling pathways. Retrograde endocannabinoid signaling is capable of decreasing synaptic strength over extended periods [23], while calcium signaling controls the release of neurotransmitters and neuronal energy metabolism [24], and phospholipase D controls neurite growth, nerve cell endocytic activity, and membrane transport [25]. The GSEA analysis supported the importance of inflammatory pathways, including cytokine-cytokine receptor interactions, TLR signaling, and NF-κB signaling pathways. These data are consistent with findings from Ren et al., who studied traumatic brain injury in humans, revealing a key role for inflammatory immune deficiencies in the context of CNS trauma [26]. Based on the above analyses, the present results support a role for the identified DE mRNAs in the pathogenesis of SCI through various mechanisms, suggesting many directions for the development of treatment to improve patient outcomes.

SCI-associated damage to the nervous system results from both primary mechanical injury and a complex cascade of secondary damage. Treatment efforts explored to date include immunoregulatory, regenerative, and neuroprotective approaches [1]. Recently, researchers have found that circRNA can protect nerve cells by targeting the expression of circRNAs in nerve cells, suggesting novel potential treatments for neurological diseases. Yao et al. demonstrated the anti-apoptotic activity of circRNA.7079 in NSC-34 cells via a mmu-miR-6953-5p-LGALS3 pathway, thereby protecting neuronal cells [27]. Liu et al. additionally found that circ_HIPK3 acts as a pathogenic inhibitor of neuronal apoptotic death following CoCl-_2_ treatment via the upregulation of DUSP19 mediated by miR-222-3p [28]. Moreover, Wu et al. determined that silencing of circRNA_01477 led to significant upregulation of miRNA 423-5p, resulting in reduced astrocyte migration and proliferation and highlighting a role for this pathway in the context of axonal regeneration in SCI [29].

The immune system-mediated inflammatory response is an important microenvironmental factor associated with the pathogenesis of SCI. The identification of critical circRNAs that can suppress inflammation has the potential to enhance angiogenic activity, prevent neuronal cell damage, and alter excitatory neurotransmitter secretion to promote healing following SCI [1]. Recent evidence suggests that circRNAs can act as sponges for miRNAs, regulating gene expression and, in turn, controlling macrophages and other cell types, suppressing inflammatory cytokine release and mitigating neuronal damage. For example, Circ_ANRIL can sponge miR-622 and can thus negatively regulate the expression of this miRNA in OGD/R-treated human microvascular endothelial cells [30]. The silencing of Circ_ANRIL can also suppress inflammation and apoptotic cell death through miR-622-dependent regulatory activity [30].

Advances in the prediction of molecular interactions using computational biology has led to the development of prediction models such as GCNCRF [31], NDALMA [32], and GCNAT [33]. These allow the efficient prediction of miRNA-circRNA/lncRNA interactions and contribute significantly to our understanding of circRNA/lncRNA functions in many diseases. Many researchers believe that computational biology is one of the important directions for researching diseases in the future. In this study, a ceRNA network specifically composed of immune inflammation-related targets was constructed by computational biology. This study is the first to our knowledge to have employed bioinformatics techniques to assess the role of circRNA-mediated ceRNA activity as a regulator of post-SCI immune inflammatory activity. It will provide potential key targets for the treatment of SCI.

Macrophages are a unique type of immune cell with important functions, which are divided into two categories in the central nervous system, namely, specific central nervous system-resident macrophages (microglia) and bone marrow-derived macrophages (BMDM) [34]. During the secondary injury of SCI, infiltrated BMDMs migrate to the injury center, while microglia derived from macrophages are distributed near the site of injury [34]. Therefore, macrophages near the center of the lesion are mainly of the BMDM type. Macrophages show dynamic changes in phenotypic morphology and function, which can be roughly divided into the M0 phenotype (resting homeostatic) to the M1 phenotype (pro-inflammatory), and the M2 phenotype (anti-inflammatory) [34]. Within hours of SCI, macrophages transform into the M1 type, releasing a large number of inflammatory factors and causing damage to neurons [34]. Therefore, during the inflammatory reaction in SCI, regulating the polarization of macrophages toward the M2 phenotype or reducing the polarization toward the M1 phenotype may be an effective method to reduce the degree of secondary SCI [35].

The present analyses highlighted the macrophage scavenger receptor 1 (Msr1) as a target in the constructed immune inflammation-associated ceRNA network. As a scavenger receptor, Msr1 plays a role in processes such as bone metabolism, host defense, regulation of inflammation, and the endocytosis of modified low-density lipoprotein [36, 37]. Guo et al. observed enhanced JNK activation in macrophages that had been activated with IL-4 when Msr1 was triggered, leading to a phenotypic shift in these cells away from an anti-inflammatory phenotype and toward a pro-inflammatory phenotype [38]. Msr1 can also facilitate macrophage transformation into foam cells through myelin sheath fragment phagocytosis and controls inflammatory mediator release via NF-κB signaling activity, ultimately contributing to the apoptotic death of neurons after SCI [39]. Govaere et al. found that under the induction of saturated fatty acids, MSR1 can induce macrophage proinflammatory reaction through the JNK signaling pathway, and aggravate non-alcoholic fatty liver disease (NAFLD) [40]. Researchers used monoclonal antibodies to target MSR1 in obesity-related NAFLD mouse models and found that foam macrophages and inflammatory factors were significantly reduced [40]. In addition, it has been reported in many studies that rno-miR-667-5p participates in multiple ceRNA mechanisms to regulate disease development. According to the study by Feng et al., bone marrow mesenchymal stem cells inhibit liver fibrosis through a lnc-BIHAA1/rno-miR-667-5p-mediated mechanism [41]. Overexpression of rno-miR-667-5p in hepatic stellate cell aggravates the progression of fibrosis [41]. CircPan3 targets miR-667-5p to increase ghrelin synthesis and chondrocyte autophagy, which protects against osteoporosis injury, according to Zeng et al. [42]. Li et al. reported that in the pathogenesis of diabetic nephrosis, circ_0000181 promoted the activation of the NLRC4 inflammasome through competitive sponging of miR-667-5p, promoted the release of IL-1β and IL-18, and caused pyroptosis of MRTEC cells [43]. Li et al. reported that caspase-1 inhibition (Vx-765) can promote microglia to change from M1 type to M2 type, reduce the secretion of inflammatory factors, and achieve the effect of protecting neurons in the process of the mouse model of ischemic stroke [44]. Therefore, miR-667-5p may be involved in regulating the polarization of macrophages. Accordingly, the RNO_CIRCpedia_4214/RNO-miR-667-5p/Msr1 axis was chosen as a focus for macrophage polarization experimental verification. The important condition that a circRNA can play the ceRNA mechanism is that it is mainly located in the cytoplasm [45]. We found RNO_CIRCpedia_4214 is mainly located in the cytoplasm through FISH experiment, so it may play a role through ceRNA mechanism. Silencing the expression of this circRNA in macrophages led to upregulation of RNO-miR-667-5p, while Msr1 was downregulated, which was consistent with the bioinformatics results predicting the regulatory axis of RNO_CIRCpedia_4214. Dual-luciferase reporter assays confirmed that RNO_CIRCpedia_4214 can bind directly to RNO-miR-667-5p. Moreover, RNO_CIRCpedia_4214 silencing was found to promote M2 macrophage polarization. Thus, these results provide preliminary support for a link between the RNO_CIRCpedia_4214/RNO-miR-667-5p/Msr1 axis and the immune inflammatory response.

This analysis has a number of limitations. For one, the sample size was relatively limited, and further large-scale microarray analyses have the potential to yield more comprehensive mRNA and circRNA expression profiles. Second, SCI is a dynamic disease process, and patterns of gene expression are thus in constant flux. Accordingly, efforts to assess mRNA and circRNA levels at different time points may yield additional novel insights. Third, our experiments were limited to the experimental validation of animal samples rather than human samples. Fourth, the functional analyses conducted were only completed at the cellular level, highlighting the need for in vivo verification of these results in a more complex environment. Accordingly, future research will focus on the RNO_CIRCpedia_4214/RNO-miR-667-5p/Msr1 axis in vivo*.* Lastly, individual circRNAs can bind several miRNAs, which can, in turn, bind several mRNAs [46]. The blocking of one circRNA is thus not sufficient to fully confirm the proposed network relationships, given that such regulatory activity may be highly complex, necessitating more systematic studies, like rescue experiments and RNA pull-down assay, etc.

In this study, bioinformatics analysis showed that many mRNAs and circRNAs were abnormally expressed in the context of SCI pathogenesis. Through the construction of an immune inflammatory-related ceRNA network, interactions among these genes and ncRNAs were further explored to better understand the roles that these targets may play in this deleterious process. RNO_CIRCpedia_4214 may sponge RNO-miR-667-5p to stimulate Msr1 and promotes macrophage M2-like polarization, thus constructing RNO_CIRCpedia_4214/RNO-miR-667-5p/Msr1 axis in SCI.

## Supplementary Information

**Additional file 1: Supplementary Table 1.** Primers for qRT-PCR. **Supplementary Table 2.** Probe sequence of RNO_CIRCpedia_4214. **Supplementary Table 3.** The top 15 most upregulated GSEA approach in SCI.

**Additional file 2: Supplemental Figure 1.** Untrimmed original image of Fig. 7E. Different lanes 1-3 are siNC, siNC+LPS, siRNA+LPS, respectively. The image marked in red box is original image of Fig. 7E. Due to improper operation in clipping the image, the images of the original blots of Arg1 in Figure ① is missing. We will pay attention to the integrity of the original image in future work.

## Data Availability

The original datasets in this study are stored in the GEO database of the National Biotechnology Information Center (NCBI) under accession number GSE210609.

## References

[1] Anjum A, Yazid MDI, FauziDaud M, Idris J, Ng AMH, SelviNaicker A, Ismail OHR, Athi Kumar RK, Lokanathan Y (2020). Spinal cord injury: pathophysiology, multimolecular interactions, and underlying recovery mechanisms. Int J Mol Sci.

[2] Fan B, Wei Z, Yao X, Shi G, Cheng X, Zhou X, Zhou H, Ning G, Kong X, Feng S (2018). Microenvironment imbalance of spinal cord injury. Cell Transplant.

[3] Khorasanizadeh M, Yousefifard M, Eskian M, Lu Y, Chalangari M, Harrop JS, Jazayeri SB, Seyedpour S, Khodaei B, Hosseini M, et al. Neurological recovery following traumatic spinal cord injury: a systematic review and meta-analysis. J Neurosurg Spine. 2019;30(5):683-99. 10.3171/2018.10.SPINE18802 30771786

[4] O’Shea TM, Burda JE, Sofroniew MV (2017). Cell biology of spinal cord injury and repair. J Clin Invest.

[5] Santer L, Bär C, Thum T (2019). Circular RNAs: a novel class of functional RNA molecules with a therapeutic perspective. Mol Ther.

[6] Qu S, Yang X, Li X, Wang J, Gao Y, Shang R, Sun W, Dou K, Li H (2015). Circular RNA: a new star of noncoding RNAs. Cancer Lett.

[7] Chen J, Chen T, Zhu Y, Li Y, Zhang Y, Wang Y, Li X, Xie X, Wang J, Huang M (2019). circPTN sponges miR-145-5p/miR-330-5p to promote proliferation and stemness in glioma. J Exp Clin Cancer Res.

[8] Chen W, Wang H, Feng J, Chen L (2020). Overexpression of circRNA circUCK2 attenuates cell apoptosis in cerebral ischemia-reperfusion injury via miR-125b-5p/GDF11 signaling. Mol Ther Nucleic Acids.

[9] Ma N, Pan J, Wen Y, Wu Q, Yu B, Chen X, Wan J, Zhang W (2021). circTulp4 functions in Alzheimer’s disease pathogenesis by regulating its parental gene, Tulp4. Mol Ther.

[10] Liu Q, Li Q, Zhang R, Wang H, Li Y, Liu Z, Xie W, Geng D, Wang L (2022). circ-Pank1 promotes dopaminergic neuron neurodegeneration through modulating miR-7a-5p/α-syn pathway in Parkinson’s disease. Cell Death Dis.

[11] Ye X, Chen Y, Wang J, Chen J, Yao Y, Wang L-L, Zhao F (2021). Identification of circular RNAs related to vascular endothelial proliferation, migration, and angiogenesis after spinal cord injury using microarray analysis in female mice. Front Neurol.

[12] Tong D, Zhao Y, Tang Y, Ma J, Wang Z, Li C (2021). Circ-Usp10 promotes microglial activation and induces neuronal death by targeting miRNA-152-5p/CD84. Bioengineered.

[13] Wang W, He D, Chen J, Zhang Z, Wang S, Jiang Y, Wei J (2021). Circular RNA Plek promotes fibrogenic activation by regulating the miR-135b-5p/TGF-βR1 axis after spinal cord injury. Aging (Albany NY).

[14] Cao J, Tang T, Tan J, Chen Q, Yuan J, Li T, Cheng X (2023). Expression profiles of long noncoding RNAs and messenger RNAs in a rat model of spinal cord injury. Comput Math Methods Med.

[15] Tay Y, Kats L, Salmena L, Weiss D, Tan SM, Ala U, Karreth F, Poliseno L, Provero P, Di Cunto F (2011). Coding-independent regulation of the tumor suppressor PTEN by competing endogenous mRNAs. Cell.

[16] Fang M, Dai H, Yu G, Gong F (2005). Gene delivery of SOCS3 protects mice from lethal endotoxic shock. Cell Mol Immunol.

[17] He AT, Liu J, Li F, Yang BB (2021). Targeting circular RNAs as a therapeutic approach: current strategies and challenges. Signal Transduct Target Ther.

[18] Chen X, Yang T, Wang W, Xi W, Zhang T, Li Q, Yang A, Wang T (2019). Circular RNAs in immune responses and immune diseases. Theranostics.

[19] Wang Y, Mo Y, Peng M, Zhang S, Gong Z, Yan Q, Tang Y, He Y, Liao Q, Li X (2022). The influence of circular RNAs on autophagy and disease progression. Autophagy.

[20] Wang C-C, Han C-D, Zhao Q, Chen X (2021). Circular RNAs and complex diseases: from experimental results to computational models. Brief Bioinform.

[21] Ge E, Yang Y, Gang M, Fan C, Zhao Q (2020). Predicting human disease-associated circRNAs based on locality-constrained linear coding. Genomics.

[22] Zhao Q, Yang Y, Ren G, Ge E, Fan C (2019). Integrating bipartite network projection and KATZ measure to identify novel CircRNA-disease associations. IEEE Trans Nanobioscience.

[23] Castillo PE, Younts TJ, Chávez AE, Hashimotodani Y (2012). Endocannabinoid signaling and synaptic function. Neuron.

[24] Brini M, Calì T, Ottolini D, Carafoli E (2014). Neuronal calcium signaling: function and dysfunction. Cell Mol Life Sci.

[25] Kanaho Y, Funakoshi Y, Hasegawa H (2009). Phospholipase D signalling and its involvement in neurite outgrowth. Biochim Biophys Acta.

[26] Ren D, Chen W, Cao K, Wang Z, Zheng P (2020). Expression profiles of long non-coding RNA and messenger RNA in human traumatic brain injury. Mol Ther Nucleic Acids.

[27] Yao Y, Wang J, He T, Li H, Hu J, Zheng M, Ding Y, Chen Y-Y, Shen Y, Wang L-L (2020). Microarray assay of circular RNAs reveals cicRNA.7079 as a new anti-apoptotic molecule in spinal cord injury in mice. Brain Res Bull.

[28] Liu Y, Ao S, Zhang H, Zhang Y, Wang Y, Yang X, Leng H (2021). Circ_HIPK3 alleviates CoCl-induced apoptotic injury in neuronal cells by depending on the regulation of the miR-222-3p/DUSP19 axis. Biochem Biophys Res Commun.

[29] Wu R, Mao S, Wang Y, Zhou S, Liu Y, Liu M, Gu X, Yu B (2019). Differential circular RNA expression profiles following spinal cord injury in rats: a temporal and experimental analysis. Front Neurosci.

[30] Jiang S, Zhao G, Lu J, Jiang M, Wu Z, Huang Y, Huang J, Shi J, Jin J, Xu X (2020). Silencing of circular RNA ANRIL attenuates oxygen-glucose deprivation and reoxygenation-induced injury in human brain microvascular endothelial cells by sponging miR-622. Biol Res.

[31] Wang W, Zhang L, Sun J, Zhao Q, Shuai J (2022). Predicting the potential human lncRNA-miRNA interactions based on graph convolution network with conditional random field. Brief Bioinform.

[32] Zhang L, Yang P, Feng H, Zhao Q, Liu H (2021). Using network distance analysis to predict lncRNA-miRNA interactions. Interdiscip Sci.

[33] Sun F, Sun J, Zhao Q (2022). A deep learning method for predicting metabolite-disease associations via graph neural network. Brief Bioinform.

[34] Zhou J, Li Z, Wu T, Zhao Q, Zhao Q, Cao Y (2020). LncGBP9/miR-34a axis drives macrophages toward a phenotype conducive for spinal cord injury repair via STAT1/STAT6 and SOCS3. J Neuroinflammation.

[35] Li X, Kang J, Lv H, Liu R, Chen J, Zhang Y, Zhang Y, Yu G, Zhang X, Ning B (2021). CircPrkcsh, a circular RNA, contributes to the polarization of microglia towards the M1 phenotype induced by spinal cord injury and acts via the JNK/p38 MAPK pathway. FASEB J.

[36] Zhao S-J, Kong F-Q, Jie J, Li Q, Liu H, Xu A-D, Yang Y-Q, Jiang B, Wang D-D, Zhou Z-Q (2020). Macrophage MSR1 promotes BMSC osteogenic differentiation and M2-like polarization by activating PI3K/AKT/GSK3β/β-catenin pathway. Theranostics.

[37] Kelley JL, Ozment TR, Li C, Schweitzer JB, Williams DL (2014). Scavenger receptor-A (CD204): a two-edged sword in health and disease. Crit Rev Immunol.

[38] Guo M, Härtlova A, Gierliński M, Prescott A, Castellvi J, Losa JH, Petersen SK, Wenzel UA, Dill BD, Emmerich CH (2019). Triggering MSR1 promotes JNK-mediated inflammation in IL-4-activated macrophages. EMBO J.

[39] Kong F-Q, Zhao S-J, Sun P, Liu H, Jie J, Xu T, Xu A-D, Yang Y-Q, Zhu Y, Chen J (2020). Macrophage MSR1 promotes the formation of foamy macrophage and neuronal apoptosis after spinal cord injury. J Neuroinflammation.

[40] Govaere O, Petersen SK, Martinez-Lopez N, Wouters J, Van Haele M, Mancina RM, Jamialahmadi O, Bilkei-Gorzo O, Lassen PB, Darlay R (2022). Macrophage scavenger receptor 1 mediates lipid-induced inflammation in non-alcoholic fatty liver disease. J Hepatol.

[41] Feng Y, Li Y, Xu M, Meng H, Dai C, Yao Z, Lin N (2022). Bone marrow mesenchymal stem cells inhibit hepatic fibrosis via the AABR07028795.2/rno-miR-667–5p axis. Stem Cell Res Ther.

[42] Zeng J, Zhang Z, Liao Q, Lu Q, Liu J, Yuan L, Liu G (2021). CircPan3 promotes the ghrelin system and chondrocyte autophagy by sponging miR-667-5p during rat osteoarthritis pathogenesis. Front Cell Dev Biol.

[43] Li Y, Yu W, Xiong H, Yuan F (2022). Circ_0000181 regulates miR-667-5p/NLRC4 axis to promote pyroptosis progression in diabetic nephropathy. Sci Rep.

[44] Li Q, Dai Z, Cao Y, Wang L (2019). Caspase-1 inhibition mediates neuroprotection in experimental stroke by polarizing M2 microglia/macrophage and suppressing NF-κB activation. Biochem Biophys Res Commun.

[45] Tay Y, Rinn J, Pandolfi PP (2014). The multilayered complexity of ceRNA crosstalk and competition. Nature.

[46] Wang W-Z, Li J, Liu L, Zhang Z-D, Li M-X, Li Q, Ma H-X, Yang H, Hou X-L (2021). Role of circular RNA expression in the pathological progression after spinal cord injury. Neural Regen Res.

