# Supplementary material for: Analysis and verification of the circRNA regulatory network RNO_CIRCpedia_ 4214/RNO-miR-667-5p/Msr1 axis as a potential ceRNA promoting macrophage M2-like polarization in spinal cord injury
Source: BMC Genomics. 2023 Apr 5;24:181. doi: 10.1186/s12864-023-09273-w (PMC10077679; doi:10.1186/s12864-023-09273-w)
Supplement: Supplementary file 1 — Additional file 1: Supplementary Table 1. Primers for qRT-PCR. Supplementary Table 2. Probe sequence of RNO_CIRCpedia_4214. Supplementary Table 3. The top 15 most upregulated GSEA approach in SCI. [file 12864_2023_9273_MOESM1_ESM.docx]

*Supporting Information*

**Supplementary Table 1 Primers for qRT-PCR.**

| ID | Primer |
| --- | --- |
| RNO_CIRCpedia_9210 | forward: 5'-ATTCGGCAGAGTCTCAGTCCA-3' |
|  | reverse: 5'-CAAGCTCTTTTCCAGCATTTCTA-3' |
| RNO_CIRCpedia_6416 | forward: 5'-ACTTTCTATCATCAAGGTCACGAGC-3' |
|  | reverse: 5'-TTCATCATCAGTCTCAGCATCCC-3' |
| RNO_CIRCpedia_4214 | forward: 5'-TGTTTGTGGTGCCTCACTCCC-3' |
|  | reverse: 5'-TGATCTCGTTGTTCTCCGCTAG-3' |
| RNO_CIRCpedia_5663 | forward: 5'-CGGAACTCCATGTACCATAATAAGC-3' |
|  | reverse: 5'-GGAACATTCAATCCCAAACACCT-3' |
| NLRP3 | forward: 5'-GTTAGTCCGCCTTTGACAGTGAT-3' |
|  | reverse: 5'-AAGCAAAGTTCCTCCAGACAGC-3' |
| Msr1 | forward: 5'-GCGAATCTACAGCAAAGCAACA-3' |
|  | reverse: 5'-TCTGTGAGTGTTCCCAGTCCTTC-3' |
| Tlr6 | forward: 5'- GTCTCCCCACTTCATCCAGA-3' |
|  | reverse: 5'- CCCACGTTTACCCTTCTCAA-3' |
| Caspase-1 | forward: 5'- ACACGTCTTGCCCTCATTATCT-3' |
|  | reverse: 5'-ATAACCTTGGGCTTGTCTTTCA-3' |
| Arg-1 | forward: 5'-TTACAAGACAGGGCTACTTTCAGG-3' |
|  | reverse: 5'-TATGATTACCTTCCCGTTTCGTT-3' |
| iNOS  miR-667-5p | forward: 5'-CTTGGAGCGAGTTGTGGATTGTT-3'  reverse: 5'-GAGGGGTAGTGATGTCCAGGAAG-3'  forward: 5'-ACACTCCAGCTGGGCGGTGCTGGTGGAGCAGT-3'  reverse: 5'-CTCAACTGGTGTCGTGGAGTCGGCAATTCAGTTGAGGTGCTCAC-3' |
| GAPDH | forward: 5'-CTGGAGAAACCTGCCAAGTATG-3' |
|  | reverse: 5'-GGTGGAAGAATGGGAGTTGCT-3' |
| U6 | forward: 5'-CTCGCTTCGGCAGCACA-3'  reverse: 5'-AACGCTTCACGAATTTGCGT-3' |

qRT-PCR: quantitative real-time PCR.

**Supplementary Table 2 Probe sequence of RNO_CIRCpedia_4214.**

| ID | Primer |
| --- | --- |
| RNO_CIRCpedia_4214 | 5'-TGGCCCTGGGTCATTATGGGAGTGA-3' |

**Supplementary Table 3. The top 15 most upregulated GSEA approach in SCI.**

| Term_Ranked | Term | ES | NES | pval | fdr | geneset_size | matched_size |
| --- | --- | --- | --- | --- | --- | --- | --- |
| 1 | Hematopoietic cell lineage(rno04640) | 0.741436042 | 2.542669542 | 0 | 0 | 87 | 83 |
| 2 | Cytokine-cytokine receptor interaction(rno04060) | 0.626762356 | 2.415612855 | 0 | 0 | 240 | 225 |
| 3 | Cell cycle(rno04110) | 0.654151802 | 2.360057341 | 0 | 0 | 123 | 116 |
| 4 | Rheumatoid arthritis(rno05323) | 0.699822089 | 2.351070418 | 0 | 0 | 81 | 76 |
| 5 | Malaria(rno05144) | 0.744116074 | 2.314612506 | 0 | 0 | 52 | 46 |
| 6 | TNF signaling pathway(rno04668) | 0.645424701 | 2.305836039 | 0 | 0 | 106 | 103 |
| 7 | Toll-like receptor signaling pathway(rno04620) | 0.660357071 | 2.300510451 | 0 | 0 | 91 | 88 |
| 8 | Leishmaniasis(rno05140) | 0.695522149 | 2.29298478 | 0 | 0 | 65 | 64 |
| 9 | Osteoclast differentiation(rno04380) | 0.637889555 | 2.289783847 | 0 | 0 | 122 | 115 |
| 10 | Tuberculosis(rno05152) | 0.612706443 | 2.280828158 | 0 | 0 | 169 | 160 |
| 11 | Inflammatiory bowel disease (IBD)(rno05321) | 0.687202192 | 2.238984773 | 0 | 0 | 59 | 55 |
| 12 | NF-kappa B signaling pathway(rno04064) | 0.639027758 | 2.203575842 | 0 | 0 | 89 | 84 |
| 13 | Th17 cell differentiation(rno04659) | 0.618507009 | 2.187020065 | 0 | 0 | 102 | 101 |
| 14 | IL-17 signaling pathway(rno04657) | 0.624445918 | 2.176336391 | 0 | 0 | 91 | 85 |
| 15 | Phagosome(rno04145) | 0.582493497 | 2.172923963 | 0 | 0 | 169 | 160 |
