# Supplementary material for: Analysis and verification of the circRNA regulatory network RNO_CIRCpedia_ 4214/RNO-miR-667-5p/Msr1 axis as a potential ceRNA promoting macrophage M2-like polarization in spinal cord injury
Source: BMC Genomics. 2023 Apr 5;24:181. doi: 10.1186/s12864-023-09273-w (PMC10077679; doi:10.1186/s12864-023-09273-w)
Supplement: Supplementary file 2 — Additional file 2: Supplemental Figure 1. Untrimmed original image of Fig. 7E. Different lanes 1-3 are siNC, siNC+LPS, siRNA+LPS, respectively. The image marked in red box is original image of Fig. 7E. Due to improper operation in clipping the image, the images of the original blots of Arg1 in Figure ① is missing. We will pay attention to the integrity of the original image in future work. [file 12864_2023_9273_MOESM2_ESM.docx]

**Supplemental Figure 1.** Untrimmed original image of Figure 7E. Different lanes 1-3 are siNC, siNC+LPS, siRNA+LPS, respectively. The image marked in red box is original image of Figure 7E. Due to improper operation in clipping the image, the images of the original blots of Arg1 in Figure ① is missing. We will pay attention to the integrity of the original image in future work.


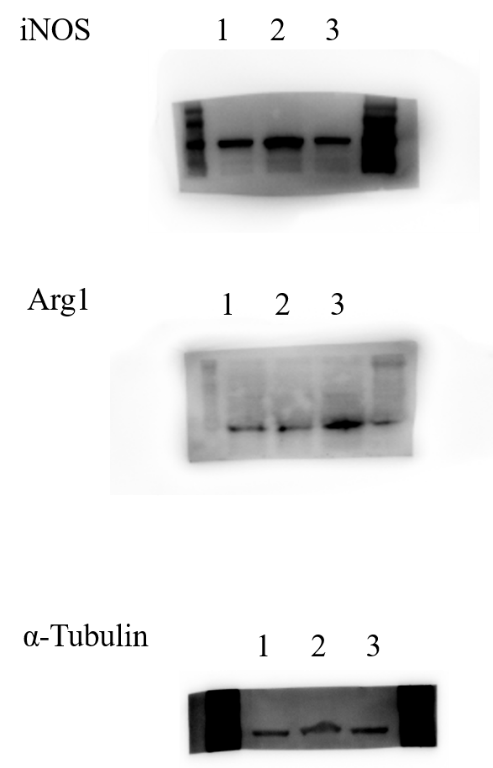

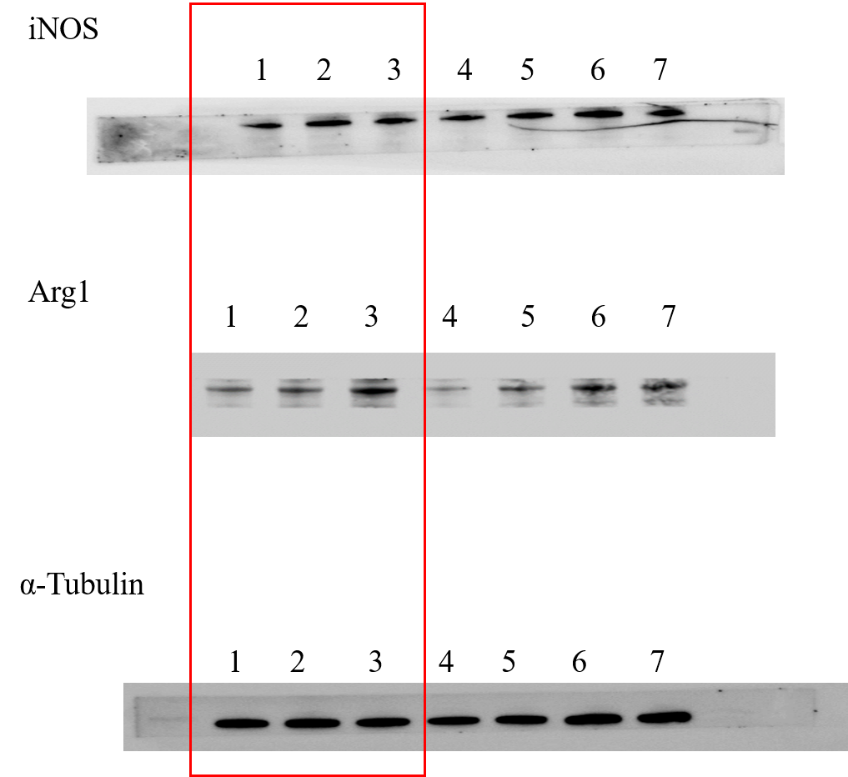
① ②


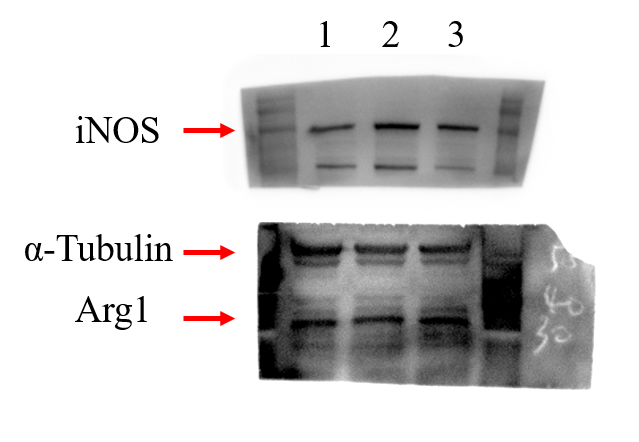
③
